# Insertion trauma of a novel inner ear catheter for intracochlear drug delivery

**DOI:** 10.3389/fvets.2024.1397554

**Published:** 2024-06-06

**Authors:** Matthias Gerlitz, Erdem Yildiz, Anselm J. Gadenstaetter, Katrin Niisuke, Sam A. Kandathil, Michael Nieratschker, Lukas D. Landegger, Clemens Honeder, Christoph Arnoldner

**Affiliations:** ^1^Department of Otorhinolaryngology, Head and Neck Surgery, Vienna General Hospital, Medical University of Vienna, Vienna, Austria; ^2^Christian Doppler Laboratory for Inner Ear Research, Department of Otorhinolaryngology, Vienna General Hospital, Medical University of Vienna, Vienna, Austria; ^3^Division of Anatomy, Center for Anatomy and Cell Biology, Medical University of Vienna, Vienna, Austria

**Keywords:** drug delivery, histology, inner ear catheter, pig, structure preservation

## Abstract

**Introduction:**

Even with recent research advances, effective delivery of a compound to its target cells inside the inner ear remains a challenging endeavor due to anatomical and physiological barriers. Direct intracochlear drug administration with an inner ear catheter (IEC) aims to overcome this obstacle and strives to provide a safe and efficient way for inner ear pharmacotherapy. The goal of this study was to histologically and audiologically evaluate the traumatic properties of a novel IEC for intracochlear drug delivery in a large animal model.

**Methods:**

Seven inner ears of piglets that had undergone intracochlear fluorescein isothiocyanate dextran application via an IEC (*n* = 4) or round window membrane (RWM) puncture with a needle (*n* = 3) followed by sequential apical perilymph sampling were histologically analyzed. Additionally, obtained objective auditory compound action potential and cochlear microphonic measurements were compared. Cochlear cryosections were stained using hematoxylin and eosin, and preservation of inner ear structures was investigated. Moreover, one cochlea was methylmethacrylate-embedded and analyzed with the IEC *in situ*.

**Results:**

Histological evaluation revealed an atraumatic insertion and subsequent compound application in a majority of IEC-inserted inner ears. Click cochlear compound action potential (CAP) shifts in the IEC groups reached a maximum of 5 dB (1.25 ± 2.5 dB) post administration and prior to perilymph sampling. In comparison, application by RWM puncture generated a maximum click CAP hearing threshold shift of 50 dB (23.3 ± 23.1 dB) coinciding with coagulated blood in the basal cochlear turn in one specimen of the latter group. Furthermore, *in situ* histology showed an atraumatic insertion of the IEC demonstrating preserved intracochlear structures.

**Conclusion:**

The IEC appears to be a promising and efficient way for inner ear drug delivery. The similarities between the porcine and human inner ear enhance the clinical translation of our findings and increase confidence regarding the safe applicability of the IEC in human subjects.

## 1 Introduction

With a predicted number of 2.5 billion people being affected in 2050, nearly 25% of the world’s population is expected to acquire some form of hearing loss in the coming decades (1). This rising tendency among all societal groups calls for safe and effective strategies to attenuate the projected outgrowth (2). Aside from hearing aids and the exceptionally successful cochlear implant, which represents today’s most frequently deployed neuroprosthesis and the gold standard to address profound hearing loss (3), therapeutic options are limited and especially specific pharmacological interventions for inner ear therapy are scarce. While corticosteroids have been used for many otologic indications with varying success rates, only a single drug, namely sodium thiosulfate, is currently approved for the prevention of cisplatin-induced hearing loss (4).

Apart from the constrained therapeutic options available today, the effective delivery of a drug to its designated tissue, i.e., typically the sensory cells of the inner ear, remains challenging. Drawbacks of systemic application include the first-pass effect and a selectively permeable blood-labyrinth barrier, often resulting in the need of high drug dosages to reach therapeutic concentrations within the cochlea. Thus, systemic drug application is often accompanied by adverse effects (5). Local delivery, as commonly performed via an intratympanic injection, relies on passive diffusion of the applied compound through the membranes of the round and oval window and is therefore heavily influenced by the solution’s molecular properties (6). Moreover, drug diffusion to the middle and apical regions of the cochlea is limited after intratympanic injection and drainage of applied drugs through the Eustachian tube must be kept in mind (7).

Due to advances in delivery technologies over the last years, direct intracochlear drug application has gained importance (8). Sustained release of a certain compound can be achieved using cochlear implants as drug carriers for continuous intrascalar delivery (9–11). While a single administration of a specific solution can be achieved via direct puncture of the round window membrane (RWM), delivery efficiency varies and can be inefficient due to fluid efflux at the injection site (12–14).

Recent studies have successfully utilized an inner ear catheter (IEC) as a novel drug delivery tool (15–17). The silicone-based IEC possesses dimensions comparable to those of a cochlear implant and features a small opening at the catheter tip enabling deep intracochlear drug administration. Steroid application via the IEC prior to cochlear implantation reduced impedances on short-term follow-up and proved to be a safe drug delivery strategy in patients with residual hearing. In human cadaveric temporal bones, fluid application using the IEC followed by cochlear implantation proved to be largely atraumatic and to preserve intracochlear structures (18).

Lately, various groups have started to investigate the pig as a novel large animal model in the field of hearing research due to the many advantages compared to commonly used alternative species like non-human primates or small animal models, such as rodents (19, 20). Strikingly, the similar length of the cochlear basilar membrane between pigs (32 mm) and humans (33.5 mm) serves to enhance the clinical translation of research findings and makes the pig well-suited for investigations of both inner ear drug delivery and cochlear implantation (20, 21).

Our group has previously investigated the applicability of the CE-marked IEC in a porcine animal model (22). Model compound application using the IEC proved to be an efficient way to increase drug concentrations for up to 24 h in the apical regions of the cochlea compared to a direct RWM puncture. Furthermore, IEC-injected cochleae also showed higher total compound concentrations in all collected perilymph samples. The aim of this study was therefore to investigate the safety of drug application via the IEC with regard to intracochlear structure preservation in the porcine inner ear applying a histological trauma scaling and using objective auditory measurements.

## 2 Materials and methods

### 2.1 Animal experiments

All animal experiments were approved by the Austrian Federal Ministry for Science and Research (BMBWF-2021-0.615.887). Details of the exact procedures can be found elsewhere (22). In short, domestic piglets of mixed strains (*Sus scrofa domesticus*) of both sexes with a weight span between 8.35 to 20 kg (12.81 ± 2.65 kg, mean ± standard deviation, SD) were used in this study. Utilizing an endaural approach, 2.5 mmoL/L fluorescein isothiocyanate dextran (FITC-d) (4,000 g/L, Sigma-Aldrich, St. Louis, Missouri, United States) was slowly injected through the round window using either the CE-marked IEC (INCAT, MED-EL, Innsbruck, Austria) or a 23-gauge needle cannula (BD Vacutainer, Becton Dickinson, Franklin Lakes, New Jersey, United States), both attached to a micropump (UMP3T-1, World Precision Instruments LLC, Sarasota, Florida, United States). In case of catheter delivery, the IEC was inserted 12.5 mm into the scala tympani measured according to the markings on the catheter relative to the RWM.

Subsequently, a controlled injection of 40 μL FITC-d solution into the scala tympani over the course of 10 min (4 μL/min) was carried out, followed by removal of the IEC afterwards. 2, 6 or 24 h after injection, sequential apical perilymph sampling (15 × 2 μL) was performed to determine the FITC-d concentration of the collected perilymph samples, and to obtain an indirect representation of the intracochlear compound distribution. After extraction of the last sample, the apical sampling hole was closed using tissue glue and all animals were euthanized by intravenous administration of pentobarbital (300 mg/kg BW).

### 2.2 Hearing measurements

Objective hearing measurements were performed using a mobile audiometry system (Duet, Intelligent Hearing Systems, Miami, Florida, United States). Acoustic stimuli were presented in decreasing steps of 10 dB beginning at 120 dB sound pressure level until no response was detected. Then, a threshold was defined in 5 dB steps around the expected hearing threshold. Intraoperative electrocochleography measurements were performed using a gold wire electrode (PromStim, MED-EL, Innsbruck, Austria) attached to the RWM for recording of acoustic compound action potentials (CAPs), for click and 20 kHz stimuli. Cochlear microphonic potentials (CMs) were measured in response to a 4 kHz acoustic stimulus with a rate of 35.1/s and 400 sweeps for both CAPs and CMs. All measurements were carried out before application of the compound and directly prior to perilymph sampling.

### 2.3 Fixation and decalcification of cochleae

Following euthanasia, cochleae were quickly extracted from the temporal bones using a hole saw. Next, removal of the stapes and of the apical sampling hole’s sealing were carried out. After careful flushing of the cochleae with 4% paraformaldehyde (PFA) using a 30-gauge needle, inner ears were placed into fresh 4% PFA solution for 24 h at 4°C. Next, cochleae were decalcified in phosphate-buffered 20% ethylenediaminetetraacetic solution (pH 7.4) at 37°C on a shaker for at least 4 weeks or until the inner ears were completely decalcified with excess bone repeatedly being cut off to accelerate decalcification time.

### 2.4 Tissue embedding

Prior to tissue embedding, excess bone was trimmed from the specimens using scalpel and forceps. Cochleae were then washed with phosphate-buffered saline (0.1 M, pH 7.4) and put into cryoprotectant medium consisting of 30% sucrose, 2% dimethyl sulfoxide, 10% glycerine, and 0.1 M phosphate buffer for 24 h at 4°C. Specimens were transferred to plastic cryomolds filled with O.C.T. compound medium (Tissue-Tek O.C.T. Compound, Sakura Finetek, Tokyo, Japan) and put on a shaker at room temperature for 4 h. Afterwards, cochleae were embedded into fresh O.C.T. compound medium and rotated overnight. Finally, samples were transferred into new cryomolds filled with O.C.T. compound medium with the round and oval windows facing upwards and the modiolus being parallel to the cutting face. Correctly oriented specimens were then transferred into dry ice for slow solidification and stored at −20°C until further processing.

### 2.5 Cryosectioning, staining, and imaging

A Leica CM3050 S cryostat (Leica Biosystems, Wetzlar, Germany) was used for the cryosectioning procedures. Chamber and chuck temperatures were set to −24°C and −20°C. Samples were allowed to stay in the cryochamber for at least 30 min prior to cutting. Inner ears were trimmed until at least two cochlear turns were visible, then cut at 8 μm thickness and mounted onto adhesion microscope slides (Epredia SuperFrost Plus Adhesion Microscope Slides, Epredia, Kalamazoo, Michigan, United States). Before storing at −20°C, slides were air-dried for at least 1 h at room temperature to ensure proper attachment of the specimen to the slide. For each cochlea, approximately 50 slides were collected, and every fifth slide stained after incubation at 60°C for 30 min using hematoxylin and eosin and mounted using quick hardening mounting medium (Eukitt, Sigma-Aldrich, St. Louis, Missouri, United States). All slides were imaged using the automated imaging system Vectra Polaris (PerkinElmer, Hopkinton, Massachusetts, United States) and cochlear cross-sections analyzed using QuPath freeware (23) applying a trauma grading scale previously proposed by Eshraghi et al. (24) (Table 1).

**Table 1:** Scale proposed by Eshraghi et al. (24) to assess the extent of cochlear trauma following insertion of an electrode array.

Grade	Extent of cochlear trauma
Grade 0	No observable trauma/intact basilar membrane
Grade 1	Touching of basilar membrane
Grade 2	Rupture of basilar membrane
Grade 3	Electrode displacement into scala vestibuli
Grade 4	Fracture of osseous spiral lamina/modiolus, tear of stria vascularis

#### 2.5.1 *In situ* histology

For histological analysis with the IEC *in situ*, one porcine cochlea was collected, inserted post-mortem, and processed as previously described (20, 25). After fixation in 4% PFA and dehydration in ethanol, embedding in methylmethacrylate was carried out. Subsequently, the specimen was ground to a mid-modiolar plane and stained with Giemsa.

## 3 Results

**Figure 1:**
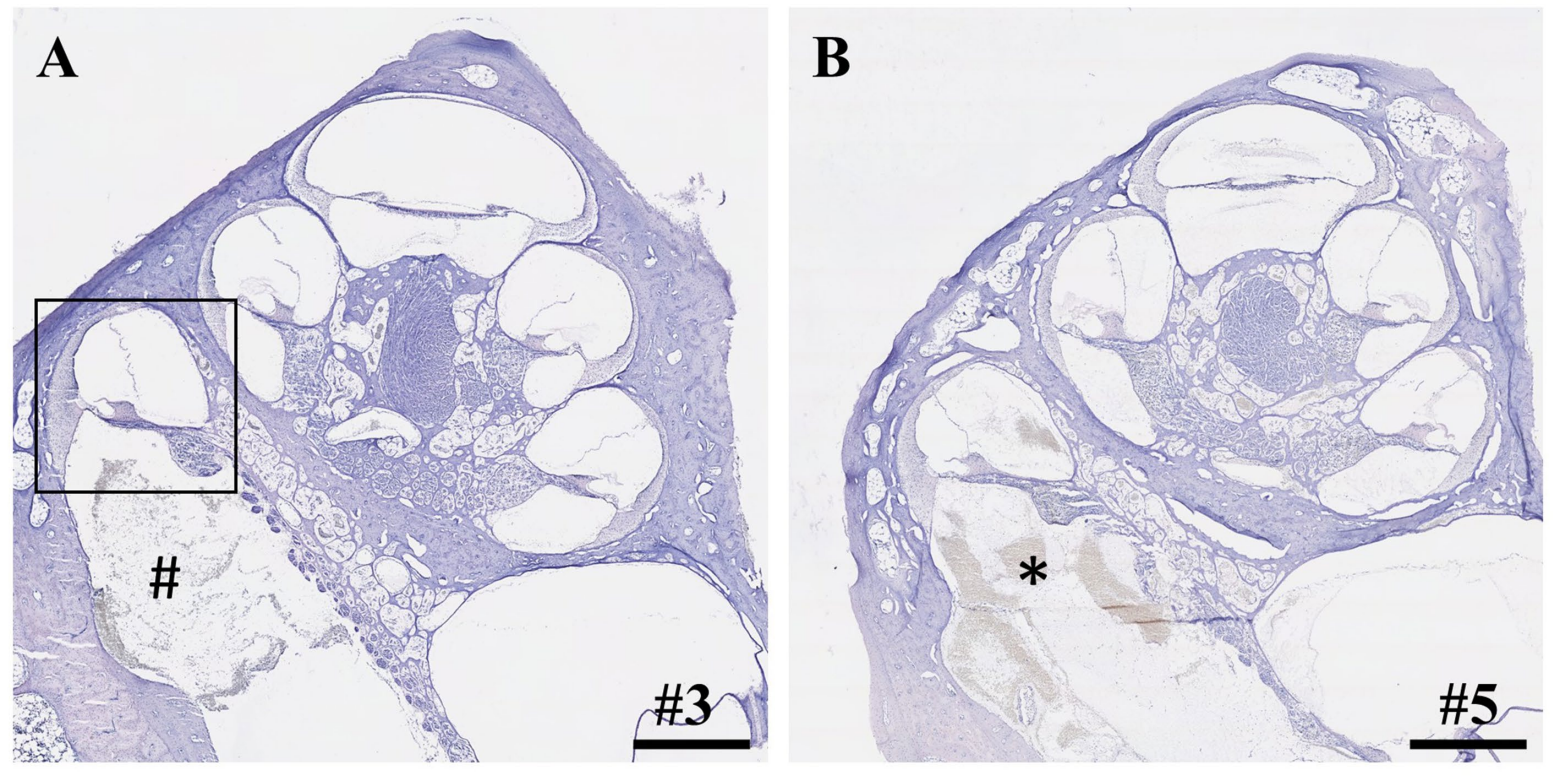
Cross-sections of cochleae after application of 40 μL FITC-d solution and apical perilymph sampling. **(A)** Cochlea after insertion of IEC, compound application, and perilymph sampling. Rectangle depicts the most basal cochlear turn with an intact basilar membrane. Hashtag shows debris inside the scala tympani. **(B)** Cochlea after compound application via direct puncture of the round window membrane and subsequent perilymph sampling. Asterisk depicts blood coagulation within the scala tympani. Numerals refer to the corresponding samples. Samples stained using hematoxylin and eosin. Scale bars = 1 mm.

Seven inner ears from three different groups (2 h IEC, 6 h IEC, 2 h RWM puncture) were randomly selected and histologically evaluated using cochlear cross-sections (Figure 1). Details are depicted in Table 2. FITC-d was applied to the left ears of piglets using the IEC in four cases and three times through a direct RWM puncture. Apical perilymph sampling took place after 2 or 6 h. After opening of the RWM, the insertion site was always clearly visible with the insertion going smoothly and without any resistance up to the intended insertion depth of 12.5 mm in all animals.

**Table 2:** Overview of the analyzed cochleae.

No.	Application technique	Sampling post application
#1	IEC	2 h
#2	IEC	2 h
#3	IEC	6 h
#4	IEC	6 h
#5	RWM puncture	2 h
#6	RWM puncture	2 h
#7	RWM puncture	2 h

IEC, inner ear catheter; RWM, round window membrane; h, hours.

**Figure 2:**
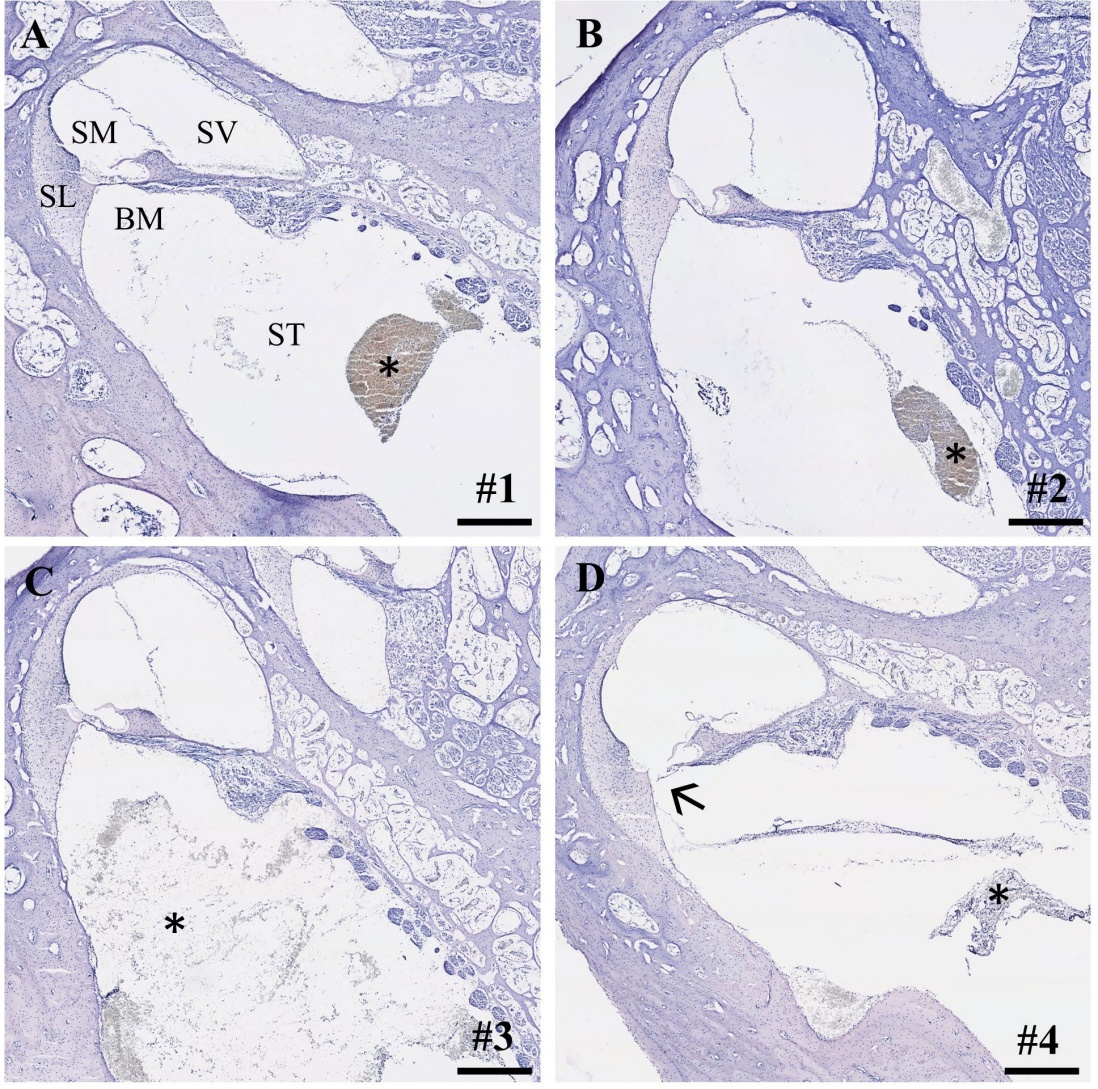
Cross-sections of the most basal cochlear turns. **(A,B)** IEC insertion and sampling after 2 h (Eshraghi scale 0) and **(C,D)** after 6 h. Arrow depicts slight displacement of the basilar membrane (sample #4, Eshraghi scale 2; **D**). Asterisks show debris in the scala tympani after IEC insertion. Numerals refer to the corresponding samples. ST, scala tympani; SV, scala vestibuli; SM, scala media; SL, spiral ligament; BM, basilar membrane. Samples stained using hematoxylin and eosin. Scale bars = 250 μm.

**Figure 3:**
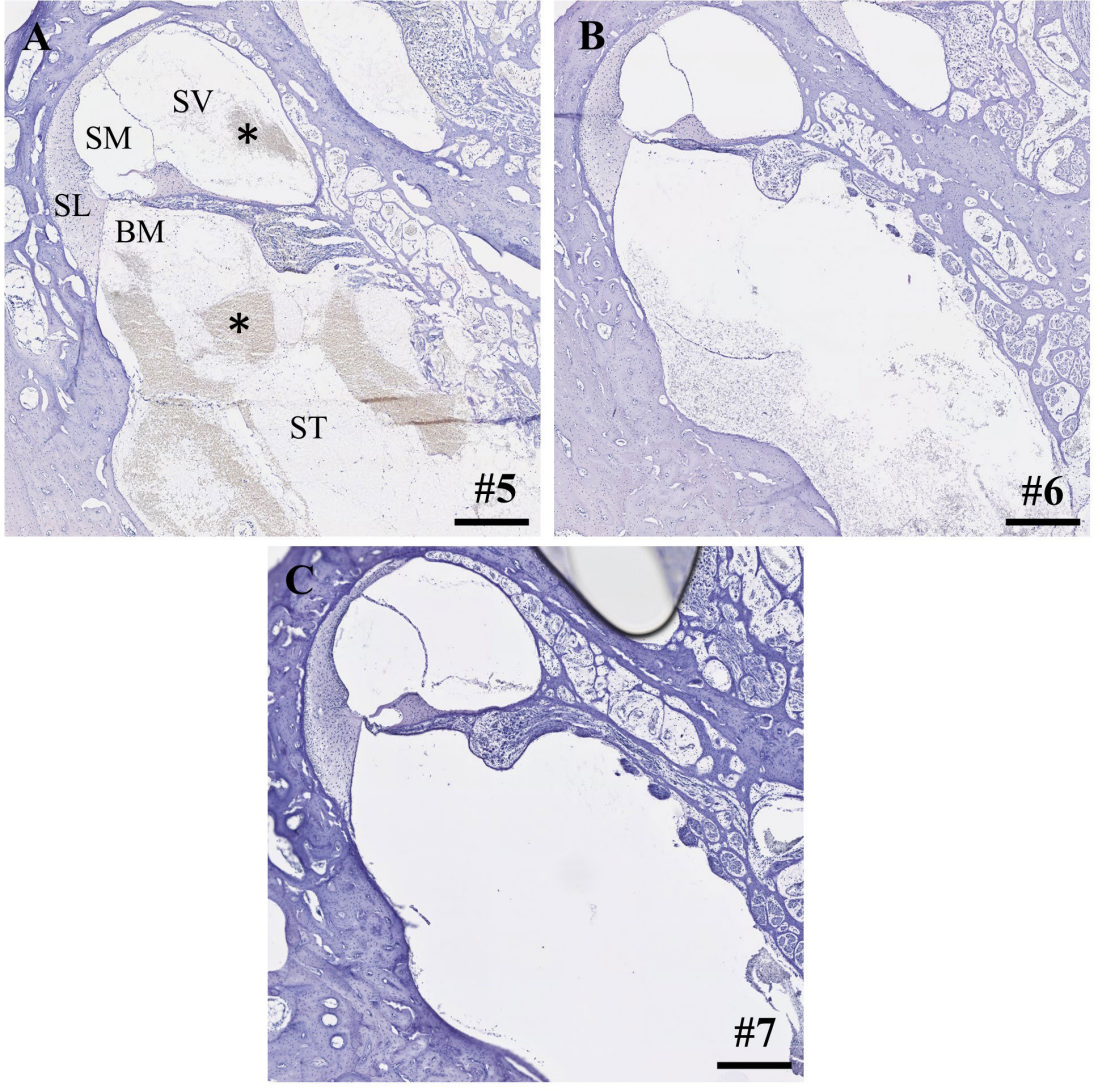
**(A-C)** Cross-sections of the most basal cochlear turns after direct round window membrane puncture, compound application and perilymph sampling after 2 h. Asterisks depict blood coagulation in the basal cochlear turn. Numerals refer to the corresponding samples. ST, scala tympani; SV, scala vestibuli; SM, scala media; SL, spiral ligament; BM, basilar membrane. Samples stained using hematoxylin and eosin. Scale bars = 250 μm.

Within the IEC groups, cochleae of animals who underwent sampling after 2 h displayed no signs of histological trauma (Eshraghi scale grade 0) in the basal cochlear turns (Figures 2A,B). Six hours after application of FITC-d and consecutive apical perilymph sampling (Figures 2C,D), one specimen showed a slight dislocation of the basilar membrane (Eshraghi scale grade 2) in the most basal cochlear turn (Figure 2D). Direct RWM puncture (2 h sampling period) did not disrupt the basal basilar membrane in the analyzed specimens (Figures 3A-C). However, blood coagulation was visible in one specimen compared to the application via the IEC (Figure 3A).

**Figure 4:**
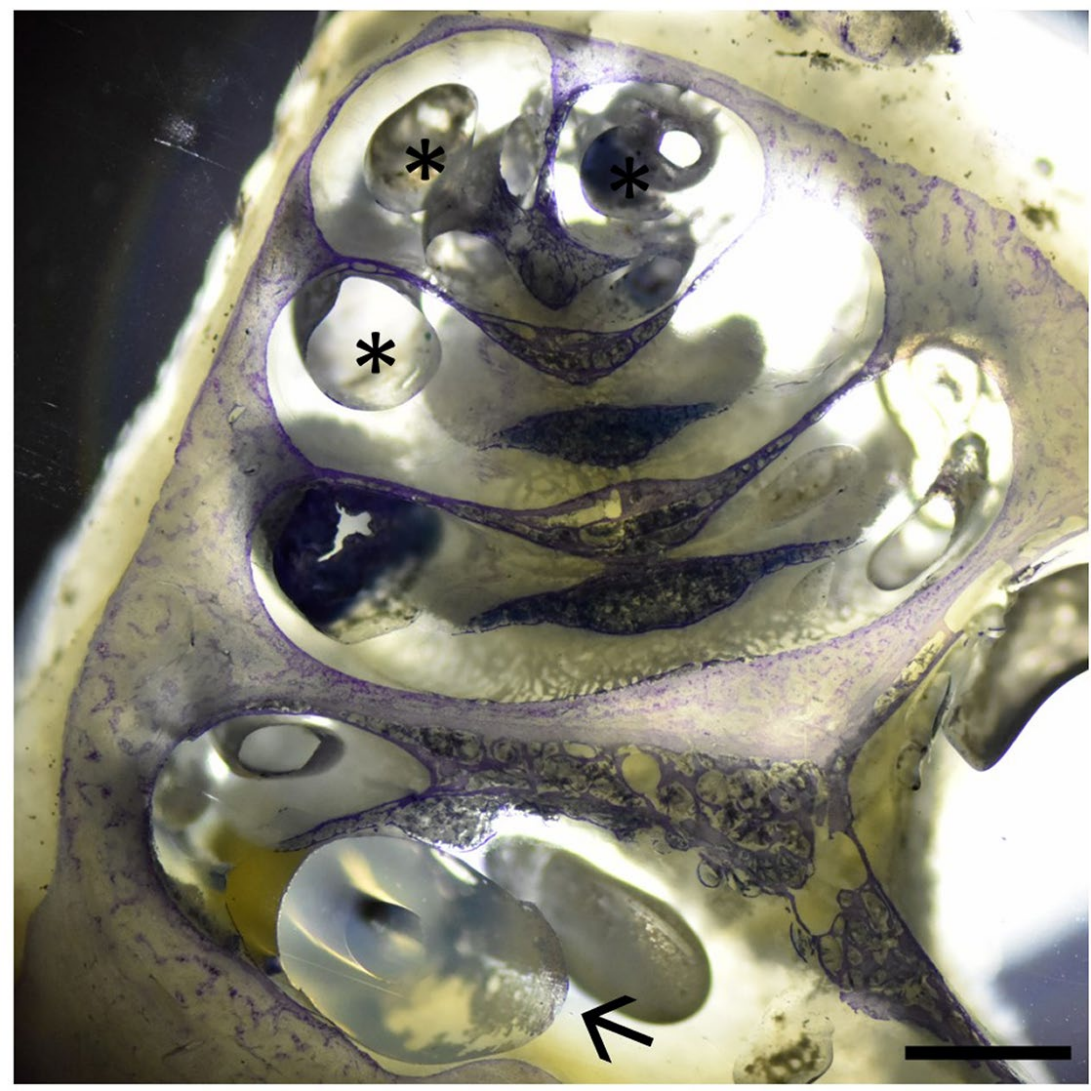
Cochlear cross-section with the IEC visible in the basal cochlear turn (arrow) touching the basilar membrane (Eshraghi scale 1). Asterisks depict embedding artifacts. Sample stained using Giemsa staining. Scale bar = 1 mm.

As stated above, one cochlea was IEC-inserted and embedded using methylmethacrylate followed by Giemsa staining. Figure 4 displays the IEC in the scala tympani of the first cochlear turn. In accordance with our histological findings, no intracochlear trauma was observed with the IEC touching the basilar membrane (Eshraghi scale grade 1).

In addition to structural damage, functional trauma following intracochlear application was assessed by audiometry. Threshold shifts were calculated between the respective measurements prior to FITC-d application and before perilymph sampling. These shifts reached a maximum of 5 dB for click (1.25 ± 2.5 dB) and 10 dB for 20 kHz CAPs (2.5 ± 5 dB) and 4 kHz CMs (3.75 ± 4.8 dB) within the IEC catheter group (Table 3). RWM puncture resulted in elevated hearing thresholds after application with a maximum shift of 50 dB (23.3 ± 23.1 dB) and 20 dB (6.6 ± 11.5 dB) for click and 20 kHz CAPs and 30 dB (25 ± 5 dB) for 4 kHz CMs, respectively (Table 3).

**Table 3:** Hearing threshold shifts after intracochlear compound application.

No.	Group	Threshold shift (dB)		
		CAP click	CAP 20 kHz	CM 4 kHz
#1	IEC 2 h	5	10	10
#2	IEC 2 h	0	0	0
#3	IEC 6 h	0	0	5
#4	IEC 6 h	0	0	0
#5	RWM puncture	50	0	25
#6	RWM puncture	10	20	20
#7	RWM puncture	10	0	30

IEC, inner ear catheter; RWM, round window membrane; h, hours; CAP, compound action potential; CM, cochlear microphonic; dB, decibel; kHz, kilohertz.

## 4 Discussion

Within our study, we provide the first histological and audiological data on previously IEC-implanted inner ears and consecutive apical perilymph sampling after different time points in a large animal model with human-like inner ear dimensions. Notably, preserved intracochlear structures after insertion and subsequent compound application utilizing the IEC were observed in a majority of the analyzed specimens. However, one cochlea showed a slight dislocation of the basilar membrane in the most basal cochlear turn after application of 40 μL FITC-d and sequential apical perilymph sampling 6 h post administration (Figure 2D). Interestingly, although a basilar membrane detachment is visible, the animal did not show a threshold shift in click or frequency-specific CAP and CM measurements prior to the start of the perilymph sampling (Table 3, sample #4). Trauma to the basilar membrane in the basal regions as observed in our study should have at least affected the high-frequency auditory CAP at 20 kHz. However, no threshold shift was observed in this specific region. This suggests that the trauma to the basilar membrane might not be a result of IEC insertion and subsequent compound application but potentially occurred later, such as during perilymph sampling or histological processing. In the course of the sampling procedure, puncturing and opening of the bony capsule may cause visible trauma to the intracochlear structures, as seen in our histological analysis. Nonetheless, the highlighted structural damage should be evident across the different frequency regions of the inner ear, not confined solely to the most basal turn, as observed in our sample. Another possible explanation is a damage following the extraction of the cochlea. After removal from the temporal bone, all cochleae were carefully flushed through the round and oval windows with 4% PFA using a 30-gauge needle. During this process, mechanically damaging the most basal region of the cochlea while inserting the needle into the scala tympani cannot be ruled out. Lastly, histological processing such as embedding and cutting of the sample can cause mechanical trauma to the various structures of the inner ear. As a consequence, we postulate that the damage of the basilar membrane observed in our study is likely not caused through insertion of the IEC, but rather occurred at a later point of the post-processing procedures.

Compound application using the IEC showed higher apical FITC-d concentrations compared to a simple RWM puncture and in contrast to an application using the IEC in combination with a stapes vent hole (22). Furthermore, IEC-injected cochleae showed higher total concentrations in all perilymph samples. Direct histological comparison of the IEC and RWM-punctured cochleae showed increased intracochlear bleeding in both scalae of the basal cochlear turn after direct RWM puncture in one analyzed specimen (Figure 3A). Accordingly, we observed a hearing threshold shift of 50 dB for click CAP and 25 dB for 4 kHz CMs in this animal, delivering a possible explanation for the deteriorated hearing responses in this animal, even though audiometric threshold shifts between the 2h groups (IEC vs. RWM puncture) did not differ significantly (22).

The proposed scale by Eshraghi et al. (24), objectifying an induced trauma to intracochlear structures after cochlear implantation, distinguishes 5 grades with more damage leading to a higher classification (Table 1). E.g., no observable macroscopic trauma results in a grade of 0, a ruptured basilar membrane corresponds to grade 2 and fracturing of the intracochlear bony structures like the spiral lamina or modiolus would be grade 4. Since our study was carried out in living animals and designed to remove the catheter after compound application, *in situ* histology with the IEC remaining inside the cochlea was not feasible in all animals. Nevertheless, we applied the commonly used Eshraghi scale in order to objectify our histological observations. Three out of four analyzed samples did not show any damage to the basilar membrane (grade 0) and one specimen displayed a membrane disruption (grade 2). However, it should be kept in mind that the trauma grades 1, namely touching of the basilar membrane, and grade 3, a dislocation of the electrode into the scala vestibuli, were not applicable and would result in either grade 2 or grade 4 trauma after removal of the catheter.

Angular insertion depths of electrode carriers, expressed in degrees around the modiolus, vary due to interindividual anatomical differences, even if arrays of the same lengths are used (26, 27). In humans, the height of the scala tympani starts to decrease at around 19 mm, reaching a critical point at approximately 23 mm, at an insertion angle of approximately 450 degrees measured from the round window (28). In the presented study, the IEC was inserted precisely 12.5 mm into the scala tympani and has thereby enabled us to achieve significantly higher apical FITC-d concentrations as well as higher overall perilymph concentrations compared to the control group (22). Considering the similarity in basilar membrane length between humans and pigs (21), insertion of the IEC in human cochleae can be expected to be atraumatic and lead to similar results as those shown in our large animal model.

The IEC is already CE-marked and available for clinical application in Europe and several countries on other continents, and its clinical applicability and safety have recently been shown both in preclinical and clinical studies (16–18). Prenzler et al. (16) used the IEC for deep intracochlear injections of triamcinolone prior to cochlear implantation resulting in significantly reduced impedances on short-term follow up. Further, steroid application using the IEC followed by cochlear implantation in patients with residual hearing turned out to be a safe and atraumatic drug delivery strategy to the inner ear, since no difference in residual hearing loss between IEC-implanted and untreated controls could be observed (17). However, it should be kept in mind that potential trauma caused by the insertion of the IEC to the basal cochlear turn may not manifest as a hearing threshold shift in those patients, since cochlear implant recipients already display little to no residual hearing in the high frequency regions. Conclusively, these recent findings lay the groundwork for using the IEC in a broad spectrum of clinical indications, for instance as a treatment option following RWM ruptures or as salvage therapy for severe cases of sudden idiopathic sensorineural hearing loss.

A main limitation of this study is the restricted number of inner ears included. A greater number of samples could have strengthened the hypotheses proposed in this study and provided a more distinct understanding of the traumatic effects associated with intracochlear drug delivery using the IEC or a direct RWM puncture with subsequent compound application. A follow-up study including a larger number of animals will be necessary to confirm our findings. Moreover, a distinction between trauma caused by the insertion of the IEC, its removal and potential artifacts caused by and during post-processing cannot be made using the applied methodology. Therefore, future studies should aim for expanded *in situ* histology or micro-CT scanning prior to the removal of the IEC to get a detailed understanding of insertion depth angles and possible interindividual differences.

## 5 Conclusion

Our study provides the first histological data investigating the traumatic properties of IEC applicability in a large animal model. IEC insertion and subsequent intracochlear compound application could be achieved without inducing damage of the delicate cochlear structures in a majority of analyzed inner ears. Hence, we postulate that the IEC is a safe and effective device for intracochlear compound application exhibiting great potential as a drug delivery strategy for various inner ear disorders.

## Data availability statement

The original contributions presented in the study are included in the article/supplementary material, further inquiries can be directed to the corresponding author.

## Ethics statement

The animal study was approved by Austrian Federal Ministry for Science and Research (BMBWF-2021-0.615.887). The study was conducted in accordance with the local legislation and institutional requirements.

## Author contributions

MG: Conceptualization, Data curation, Investigation, Methodology, Writing – original draft, Writing – review & editing, Project administration. EY: Conceptualization, Data curation, Investigation, Methodology, Writing – review & editing. AG: Conceptualization, Data curation, Investigation, Methodology, Writing – review & editing. KN: Methodology, Writing – review & editing. SK: Methodology, Writing – review & editing. MN: Formal analysis, Writing – review & editing. LL: Formal analysis, Funding acquisition, Supervision, Validation, Writing – review & editing. CH: Formal analysis, Funding acquisition, Supervision, Validation, Writing – review & editing. CA: Conceptualization, Formal analysis, Funding acquisition, Project administration, Resources, Supervision, Validation, Writing – review & editing.
